# Fruquintinib inhibits the migration and invasion of colorectal cancer cells by modulating epithelial-mesenchymal transition via TGF-β/Smad signaling pathway

**DOI:** 10.3389/fonc.2025.1503133

**Published:** 2025-03-11

**Authors:** Qinqin Song, Hongjiao Wu, Ye Jin, Junzhi Hou, Jiawei Liu, Xuemei Zhang, Wanning Hu, Guogui Sun, Zhi Zhang

**Affiliations:** ^1^ Department of Oncology, Hebei Medical University, Shijiazhuang, China; ^2^ Affliated Tangshan Gongren Hospital, Hebei Medical University, Tangshan, China; ^3^ School of Public Health, North China University of Science and Technology, Tangshan, China; ^4^ College of Clinical Medicine, North China University of Science and Technology, Tangshan, China; ^5^ Department of Oncology, Affiliated Tangshan Gongren Hospital, North China University of Science and Technology, Tangshan, China; ^6^ Department of Immunology, Tianjin Medical University Cancer Institute and Hospital, Tianjin, China; ^7^ College of Life Science, North China University of Science and Technology, Tangshan, China; ^8^ Department of Hebei Key Laboratory of Medical-Industrial Intergration Precision Medicine, North China University of Science and Technology Affiliated Hospital, Tangshan, China

**Keywords:** fruquintinib, colorectal cancer, epithelial-mesenchymal transition (EMT), TGF-β/Smad signaling pathway, vascular endothelial growth factor Receptor (VEGFR)

## Abstract

**Background:**

Fruquintinib, a selective vascular endothelial growth factor receptor (VEGFR) inhibitor, has shown considerable efficacy in colorectal cancer (CRC) treatment. Despite its promising therapeutic effects, the precise molecular mechanisms underlying its therapeutic effects remain incompletely understood. In this study, we explored the functional roles and molecular mechanisms of fruquintinib in CRC therapy.

**Material and methods:**

Human CRC cells (HCT-116 and LOVO) were cultured and treated with fruquintinib. Cell counting kit-8 assay kit (CCK-8) and colony formation assays were performed to investigate the effects of fruquintinib on cell proliferation. Wound healing and transwell assays were conducted to explore the role of fruquintinib on migration and invasion. RNA sequencing and bioinformatics analysis was used to investigate the potential mechanism of fruquintinib in the development of CRC. Western blot was used to measure the protein level.

**Results:**

Fruquintinib significantly inhibited the proliferation, migration, and invasion of colorectal cancer cells. Bioinformatics analysis indicated that fruquintinib modulated the epithelial-mesenchymal transition (EMT) pathway, and experimental validation confirmed its regulatory effects on core EMT-associated protein biomarkers. Notably, fruquintinib treatment resulted in the upregulation of E-cadherin and the downregulation of N-cadherin, vimentin, and MMP9. Western blot analysis revealed that fruquintinib dose-dependently suppressed SMAD2/3 expression. Notably, treatment with the TGF-β receptor agonist KRFK TFA attenuated fruquintinib’s effect, reversing the upregulation of E-cadherin as well as the downregulatin of N-cadherin and SMAD2/3. Additionally, KRFK TFA partially restored CRC cell migration and invasion in transwell assays, counteracting fruquintinib’s inhibitory impact.

**Conclusion:**

These findings indicate that Fruquintinib effectively hampers the migration and invasion of CRC cells by disrupting the EMT process via the TGF-β/Smad signaling pathway. This study sheds light on the mechanisms by which fruquintinib inhibits CRC progression and underscores its potential for further clinical investigation.

## 1 Introduction

Colorectal cancer (CRC) ranks as the second most lethal cancer worldwide in terms of overall incidence (1). Metastases are responsible for the majority of cancer-related death, with approximately 15-30% of CRC patients with metastatic disease at the time of diagnosis, and an additional 20-50% developing metastases later during disease progression (2, 3). Moreover, the outcome of CRC patients remains poor, with a 5-year overall survival (OS) rate of less than 15% (4). These statistics highlight an urgent clinical need for the development of novel and effective therapeutic strategies for the treatment of CRC.

Fruquintinib is a highly selective small-molecule inhibitor of vascular endothelial growth factor receptor (VEGFR), designed to block tumor neovascular growth. *In vitro*, fruquintinib effectively inhibits VEGFR-1, -2 and -3 with half-maximal inhibitory concentration (IC50) of 33, 35 and 0.5 nmol/L, respectively, while exhibiting minimal inhibitory effects on RET, FGFR-1 and c-KIT kinases (5). *In vivo*, fruquintinib has been demonstrated to exhibit promising antitumor effects in various human tumor xenograft mouse models. For example, fruquintinib inhibits stomach cancer cell growth by 24.1% and 48.6% when dosed at 5 mg/kg or 20 mg/kg daily, respectively (5). Furthermore, a phase 3 clinical trial revealed that fruquintinib significantly improved OS time by 2.7 months and the progression-free survival (PFS) time by 1.9 months (6). This further validates its antitumor efficacy in the treatment of CRC.

VEGFR-2, one of the key molecular targets of fruquintibib, is aberrantly overexpressed in many malignant tumors and contributes to the occurrence of certain cancers (7). Recent studies demonstrated that the upregulation of VEGFR-2 was correlated with reduced membranous E-cadherin and elevated levels of mesenchymal markers, such as N-cadherin, and vimentin, thereby promoting epithelial-mesenchymal transition (EMT) (8, 9). EMT plays a prominent role in tumor initiation, development, invasion and metastasis (10). EMT can be initiated by the activation of a network of transcription factors (EMT-TFs), such as SNAIL, ZEB and TWIST families, which play pivotal roles in facilitating tumor metastasis and progression (11).

Although fruquintinib has demonstrated promising anti-tumor effects *in vivo*, the molecular mechanisms underlying its action remain poorly understood. In this study, we evaluated the antitumor effects of fruquintinib on CRC cells *in vitro* and found that it disrupted the EMT process via the TGF-β/Smad signaling.

## 2 Materials and methods

### 2.1 Chemicals and reagents

Fruquintinib was purchased from Psaitong (Beijing, China). It was dissolved in DMSO at a concentration of 10 mM, then stored at 4°C until use. The TGF-β receptor agonist KRFK TFA was obtained from MedChem Express (New Jersey, USA), prepared in DMSO at a concentration of 50 μM and stored at -20°C until use. Mouse IgG (H+L) and rabbit IgG (H+L) antibodies were purchased from Seracare (Massachusetts, USA). Anti-β-actin, anti-N-cadherin and anti-E-cadherin antibodies were purchased from GeneTex (California, USA), while anti-MMP-9 and anti-vimentin antibodies were obtained from Proteintech (Wuhan, China).

### 2.2 Cell culture

Human colon cancer cell lines (HCT-116, LOVO) were obtained from American Type Culture Collection (ATCC) (Manassas, USA). Both cell lines were cultured in Dulbecco’s modified Eagle medium (DMEM) (EallBio Life Sciences, Beijing, China), supplemented with 10% fetal bovine serum (FBS) (EallBio Life Sciences, Beijing, China) and 1% penicillin and streptomycin (P/S) (EallBio Life Sciences, Beijing, China). Cells were maintained in a humidified incubator with 5% CO_2_ at 37°C. To analyze the effects of fruquintinib on colon cancer, HCT116 and LOVO cells were treated with different concentrations of fruquintinib (0, 50, 100, 150, 200 μM).

### 2.3 Cell counting kit-8 analysis

The viability of cancer cells was detected by CCK-8 assay. HCT116 and LOVO cells (5×10^3^ cells/well) were seeded into 96-well plates. After 24 h incubation, the cells were treated with fruquintinib at various concentrations. At 24 h, 48 h and 72 h post-treatment, the medium containing fruquintinib was removed, and a 10% CCK-8 solution was then added to each well for 1 h at 37°C. Cell viability was determined by measuring the optical density (OD) at 450 nm using a microplate reader (Thermoscientific, Waltham, USA). The proliferation of fruquintinib-treated cells was normalized to that of the control cells treated with the vehicle (DMSO) only. All experiments were performed in triplicate.

### 2.4 Colony formation analysis

Long-term cell viability was determined using a colony formation assay. Cancer cells were seeded into 6-well plates for 24 h, followed by treatment with fruquintinib at various concentrations (0, 100 or 200 μM for LOVO cells; 0, 50 or 100 μM for HCT116 cells) for additional 24 h. The treated cells (2×10^3^ cells/2 ml) were then resuspended and reseeded into 6-well plate. Subsequently, the cells were incubated for 14 days. After fixation with methanol and staining with crystal violet, colonies were then observed under an inverted microscope (Nikon, Tokyo, Japan). Each experiment was performed in triplicate.

### 2.5 Wound healing assay

The cancer cell migration was evaluated by a scratch wound healing assay. LOVO and HCT116 cells were homogeneously seeded in 6-well plates (4×10^5^/well). Once the cells reached 80% confluency, a scratch was created in the center of each well using a sterile pipette tip. Following treatment with different concentrations of fruquintinib, the scratch areas were observed under a reverse biological microscope (Nikon, Tokyo, Japan) at the time point of 0h, 24 h and 48 h. The wound width was measured using ImageJ software (National Institutes of Health, Bethesda, USA) and the healing rate was calculated relative to the starting wound width at 0-h time point. Each experiment was repeated in triplicate.

### 2.6 Transwell assays

The invasion and migration capacities of LOVO and HCT116 cells were evaluated using transwell assays. Transwell plates (8 μm, JET BIOFIL, Guangzhou, China) with or without Matrigel coating (Corning, New York, USA) were used in the upper chamber, while the lower chamber was filled with 600 μL of medium containing 20% FBS. To ensure consistency in cell viability and response to drug exposure, treatments were administered when the cells reached approximately 70–80% confluency. After treatment with fruquintinib (0, 50, 100, 200 μM)/fruquintinib (100, 200 μM) and KRFK TFA (50 μM) for 24 hours, 5 × 10^4^ colon cancer cells were seeded in the upper chambers. Following incubation for 24 h (migration assay) or 48 h (invasion assay), cells that have traversed the membrane were stained with crystal violet and counted under a microscope. Each experiment was repeated three times.

### 2.7 RNA-sequencing

RNA-seq was conducted by Shanghai Zhongke New Life Biotechnology (Shanghai, China). Briefly, LOVO cells were treated with or without 100 μM or 200 μM fruquintinib for 24 h. The total RNA was extracted using Trizol reagent (Magen, Guangdong, China). The RNA Screen Tape assay was used for RNA integrity assessment on Agilent 4150 Tape Station system (Agilent Technologies Inc, Beijing, China) (RIN). RNA-seq libraries were prepared using ABclonal MRA-Seq Lib Prep Kit (ABclonal, Wuhan, China), with mRNA enriched using magnetic beads conjugated with Oligo (dT). RNA was then sequenced on an Illumina Hiseq 4000 platform (Illumina, San Diego, USA).

### 2.8 Biological function and pathway enrichment analyses

DESeq2 was used to identify differentially expressed genes (DEGs) with thresholds set at |Log2FC|≥1 and a false discovery rete (FDR) <0.05. Pathway enrichment analysis was performed using cluster Profiler (version 3.14.3) R package, based on hallmark gene sets downloaded from GSEA database (https://www.gsea-msigdb.org).

### 2.9 Western blotting

Cells were lysed by RIPA lysis reagent supplemented with Phenylmethanesulfonyl fluoride (PMSF). Proteins were separated by 10% SDS-PAGE and transferred onto PVDF membranes, which were then blocked with 5% Skim Milk (Biotopped, Beijing, China) for 3 h at room temperature. The membranes were incubated with primary antibodies overnight at 4°C, including anti-β-actin (1:10000, GeneTex, California, USA), anti-N-cadherin (1:1000; GeneTex, California, USA) and anti-E-cadherin(1:1000; GeneTex, California, USA), anti-MMP9 (1:1000; Proteintech,Wuhan, China), anti-SMAD2/SMAD3 (1:10000; GeneTex, California, USA), anti-vimentin (1:5000; Proteintech, Wuhan, China), and then with conjugated secondary antibodies [anti-mouse IgG (H+L) (1:10000; Seracare, Massachusetts, USA) or anti-rabbit IgG (H+L) (1:10000; Seracare, Massachusetts, USA) for 1 h at room temperature. Protein bands were then visualized using an enhanced chemiluminescence luminescence (ECL) kit (Applygen, Beijing, China).

### 2.10 Prediction of differential expressed transcription factors

Differentially expressed transcription factors (EMT-TFs) were identified using RNA sequencing (RNA-seq) data. Differentially expressed genes (DEGs) were filtered based on |log2FC| > 1 and adjusted *p*-value (FDR) < 0.05. The Animal TFDB (http://bioinfo.life.hust.edu.cn), Pfam, and DBD databases were used to annotate the TFs of DEGs. Following annotation, we used the TRRUST2.0 database to identify the targets of the identified transcription factors. Specifically, we focused on the well-characterized EMT-TFs, including SNAI1, SNAI2, TWIST1, TWIST2, ZEB1, and ZEB2, and identified their associated targets using Sangerbox3.0. The regulation of VIM (vimentin) and CDH1 (E-cadherin) by ZEB2, SNAI1, SNAI2, ZEB1, and TWIST1 was inferred based on these regulatory relationships and the literature.

### 2.11 Statistical analysis

Statistical analyses for the wound healing assay, colony formation, transwell assays, and western blotting were performed using Student’s t-test and one‐ or two‐way analysis of variance (ANOVA). A *P* value less than 0.05 was considered statistically significant for all tests. Data analysis was conducted using SPSS (v26.0, IBM, USA).

## 3 Results

### 3.1 Fruquintinib suppresses the proliferation of colon cancer cells

**Figure 1:**
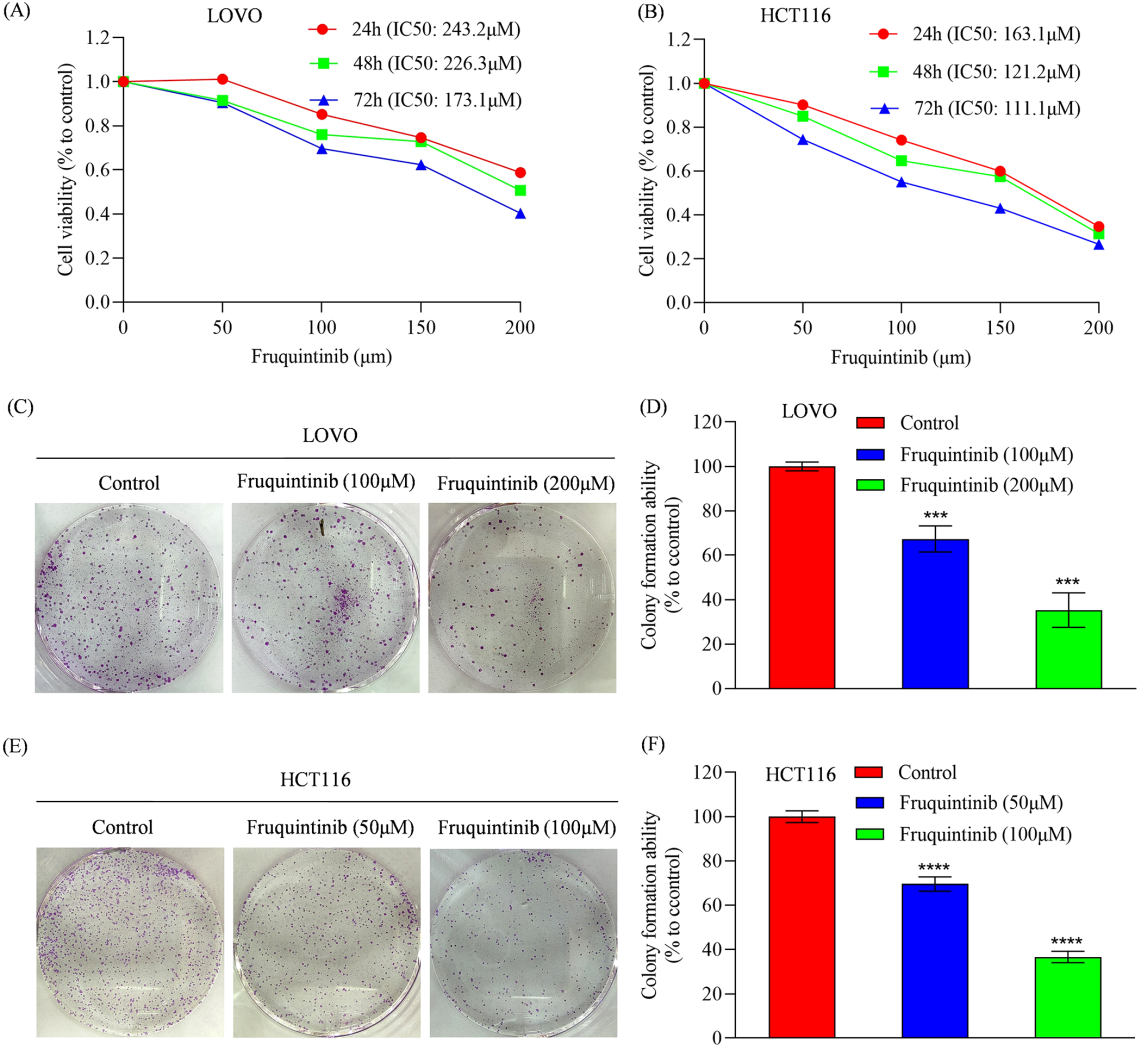
Analysis of cell proliferation in colorectal cancer cells treated with fruquintinib. **(A, B)** Cell viability measured by CCK8 assay of LOVO **(A)** and HCT116 **(B)** cells treated with the indicated concentrations of fruquintinib for 24, 48 and 72 h. **(C–F)** Representative images of colony forming assay in LOVO **(C)** and HCT116 **(E)** cells, along with their quantitative analysis **(D, F)**. Each experiment was performed in triplicate. Statistical significance compared to control group is indicated by asterisks: ***P < 0.001, ****P < 0.0001.

To determine the anti-cancer activity of fruquintinib, HCT116 and LOVO cells were treated with increasing concentrations of drug. We found that fruquintinib inhibited cell viability in a dose- and time-dependent manner. The half-maximal inhibitory concentration (IC50) values for fruquintinib in LOVO cells were 243.2, 226.3 and 173.1 μM after 24-, 48- and 72-hours incubation, respectively (**Figure 1A**). In HCT116 cells, the IC50 were 163.1, 121.2 and 111.1 μM after 24-, 48- and 72-hours incubation, respectively (**Figure 1B**). Furthermore, colony formation assays revealed that fruquintinib inhibited the proliferation of both LOVO and HCT116 cells, with higher concentration of fruquintinib demonstrating greater efficacy (**Figures 1C–F**).

### 3.2 Fruquintinib inhibits cell migration and invasion

**Figure 2:**
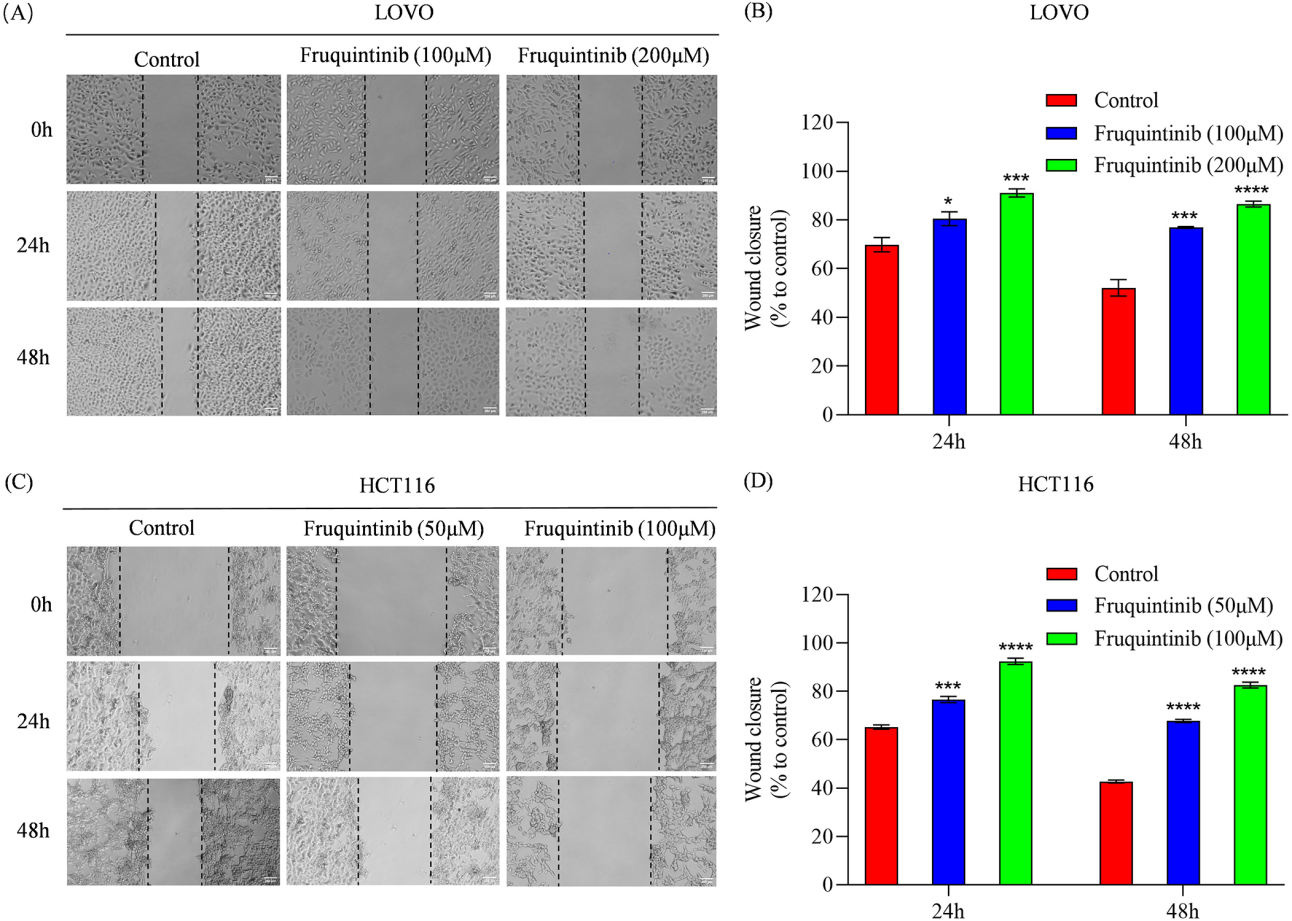
Wound healing assay of colorectal cancer cells treated with fruquintinib. **(A–D)** Wound healing assay of LOVO **(A)** and HCT116 **(C)** cells with fruquintinib treatment and their quantification (**B, D**, respectively). The images (magnification: 40×) were captured at 0, 24, 48 and 72 h after treatment. Each experiment was performed in triplicate. Statistical significance compared to control group is indicated by asterisks: *P < 0.05, ***P < 0.001, ****P < 0.0001.

**Figure 3:**
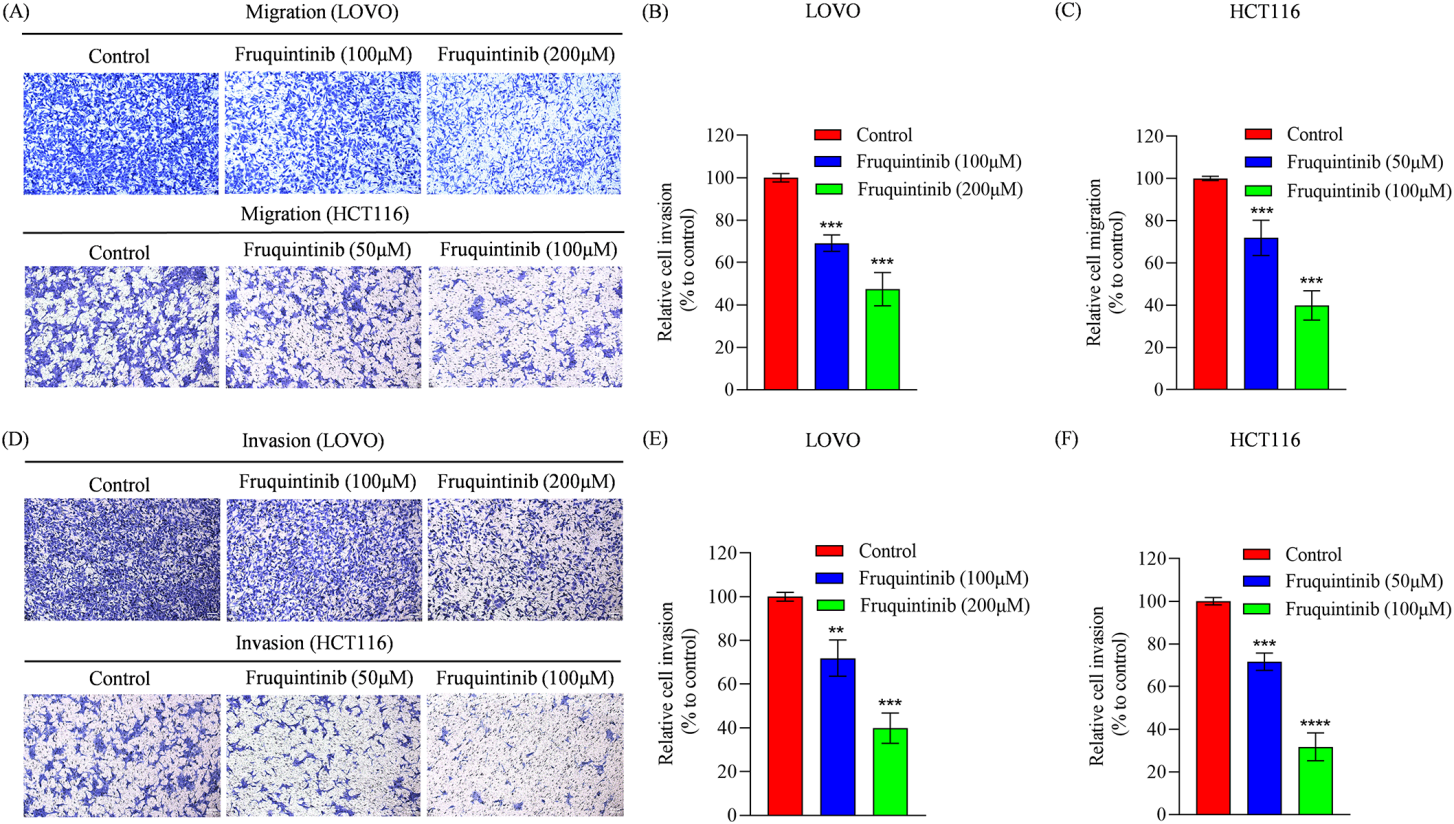
The migration and invasion analysis of CRC cells treated with fruquintinib. **(A-C)** Cell migration determined using a transwell assay following the treatment of fruquintinib in LOVO and HCT116 cells, with quantitative analysis. Magnification: 200×. **(D-F)** Cell invasion evaluated using a transwell assay following the treatment of fruquintinib in LOVO and HCT116 cells, with quantitative analysis. Magnification, 200×. The migration experiment was performed after 24 hours of treatment, while the invasion assay was performed after 48 hours of treatment. Each experiment was repeated three times. Statistical significance compared to the control group is indicated by asterisks, **P < 0.01, ***P <0.001, ****P < 0.0001.

To assess the effect of fruquintinib on the metastatic potential of CRC cells, wound healing and transwell assays were performed. In the wound healing assay, fruquintinib treatment markedly retarded the motility of CRC cells in a concertation-dependent manner (**Figures 2A–D**). Similarly, transwell assays demonstrated that fruquintinib significantly inhibited the migration and invasion capacity of the cells (**Figures 3A, D**) in a dose-dependent manner (**Figures 3B, C, E, F**).

### 3.3 Pathway analysis associated with fruquintinib targets

**Figure 4:**
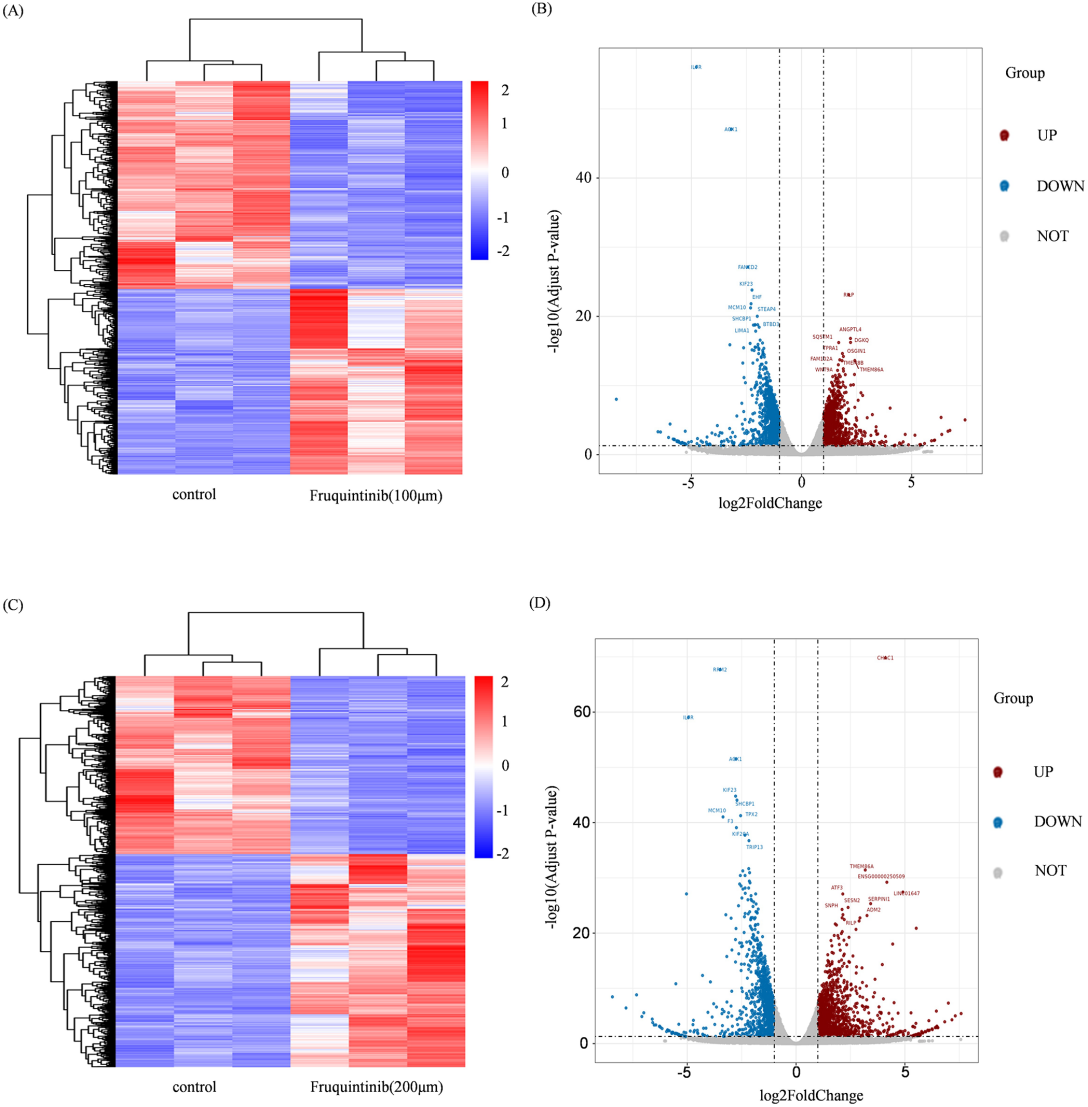
Changes in gene expression in CRC cells treated with fruquintinib. **(A, C)** The cluster heatmap of the DEGs, with a blue-to-red colour scale indicating log2 FPKM value. **(B, D)** The volcano plot of the DEGs. The blue and red indicates the down and upregulated DEGs, respectively. DEGs, differentially expressed genes; FPKM, Fragments per kilobase of transcript per million mapped reads.

**Figure 5:**
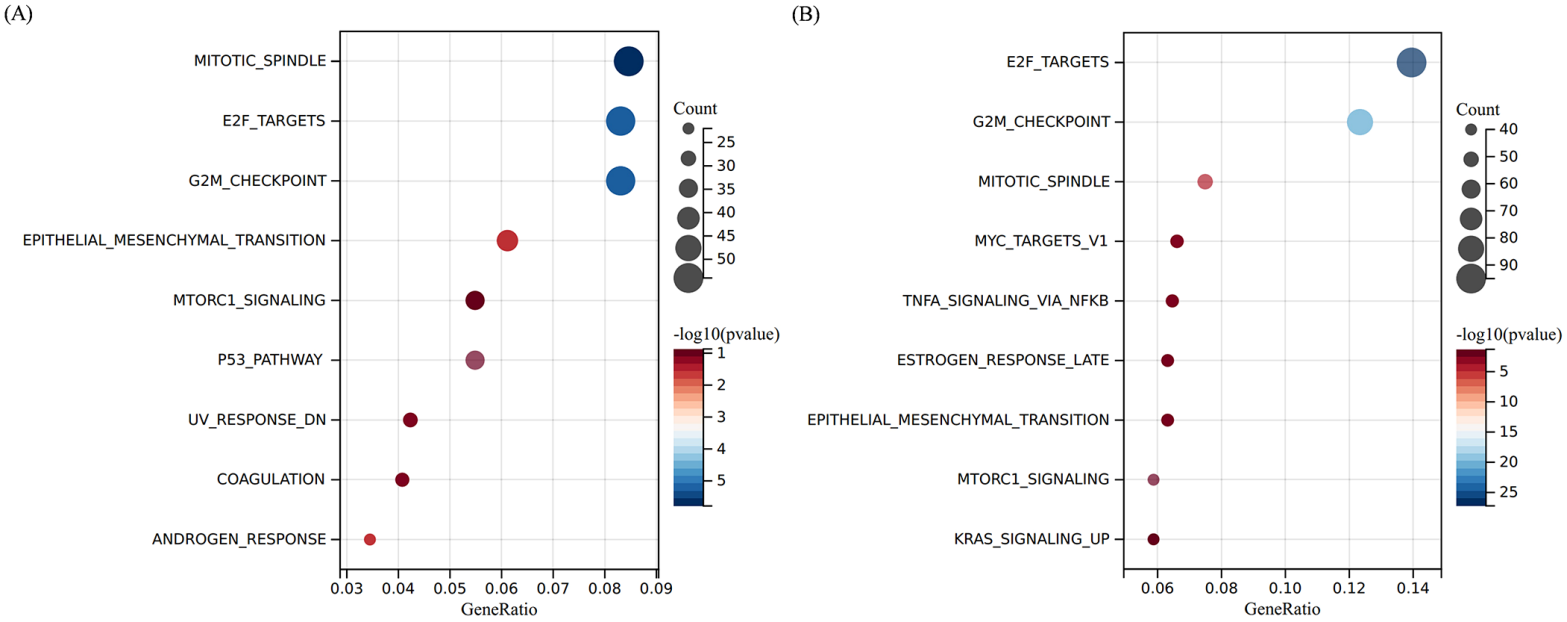
Pathway enrichment analyse of the DEGs. **(A, B)** The top 10 enriched pathways of the DEGs regulated by 100 μM **(A)** or 200 μM **(B)** fruquintinib. The colour gradient from blue to red represents -log10 (*q* value).

To identify the molecular targets regulated by fruquintinib, we conducted an RNA-seq assay using LOVO cell lines. The processed RNA sequencing data are presented in **Supplementary Table 1**. This assay revealed 1,193 and 1,489 upregulated genes, as well as 1,339 and 1,244 downregulated genes, in cells treated with 100 μM or 200μM fruquintinib, respectively (**Figures 4A–D**). Pathway enrichment analysis of the differentially expressed genes (DEGs) showed that these genes were primarily involved in mitotic spindle pathway, E2F targets pathway, G2M checkpoint pathway, epithelial mesenchymal transition (EMT) pathway and mTORC1 signaling pathway (**Figures 5A, B**).

### 3.4 Effect of fruquintinib on EMT

**Figure 6:**
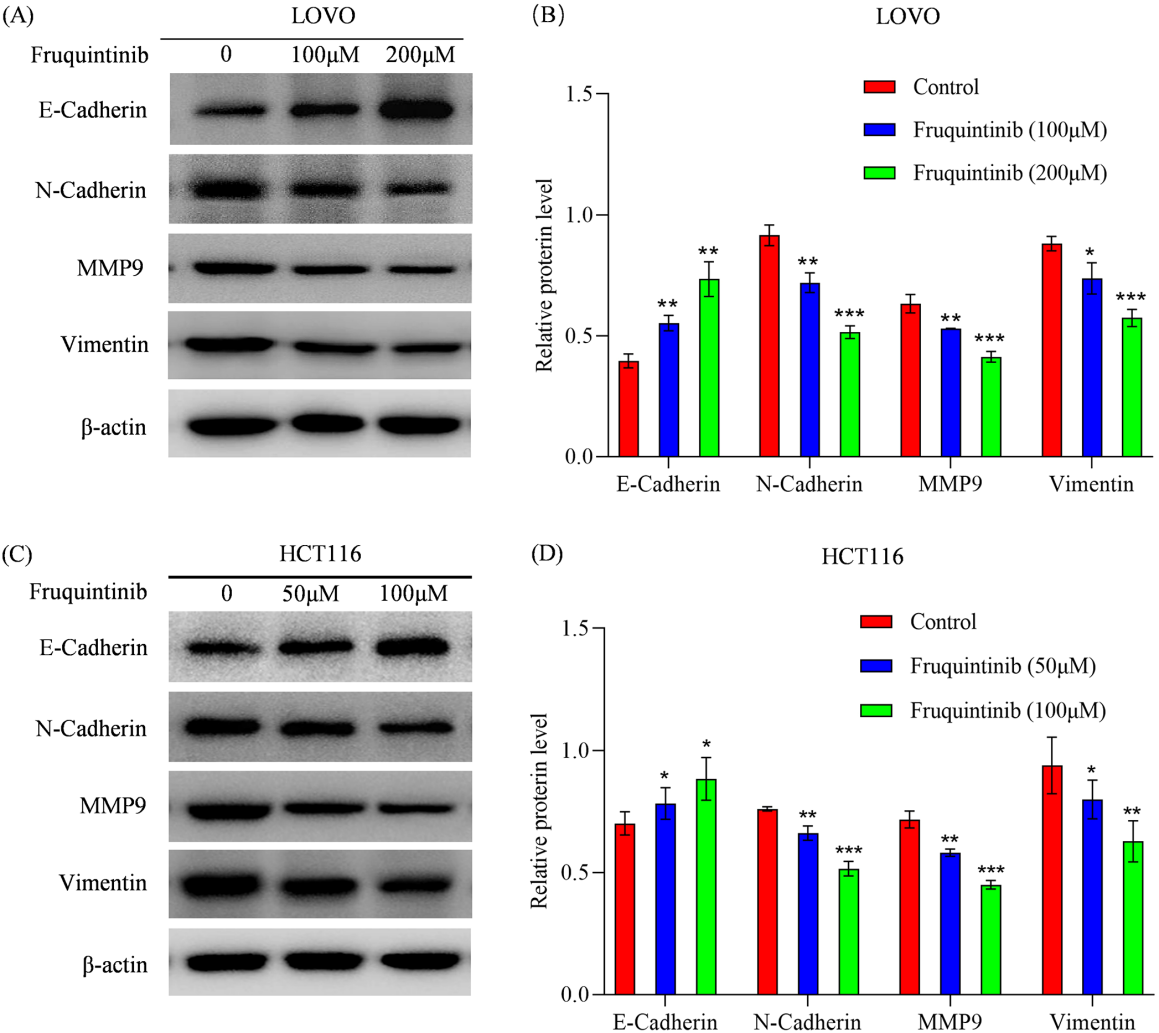
Fruquintinib inhibits the activation of EMT pathway. **(A–D)** Protein expression of key members of the EMT signaling pathway following fruquintinib treatment. Each experiment was repeated three times. Statistical significance compared to the control group is indicated by asterisks, *P < 0.05, **P < 0.01, ***P < 0.001.

To investigate whether the effects of fruquintinib on the migration and invasion of colon cancer cells are associated with EMT, we detected the expression of N-cadherin, E-cadherin, MMP-9 and vimentin following treatment with different concentrations of fruquintinib. We found that fruquintinib treatment led to a dose-dependent upregulation of E-cadherin, and a pronounced downregulation of N-cadherin, MMP-9 and vimentin (**Figure 6**).

### 3.5 Fruquintinib inhibits CRC cell migration and invasion via a TGFβ-SMAD2-3-4 pathway

**Figure 7:**
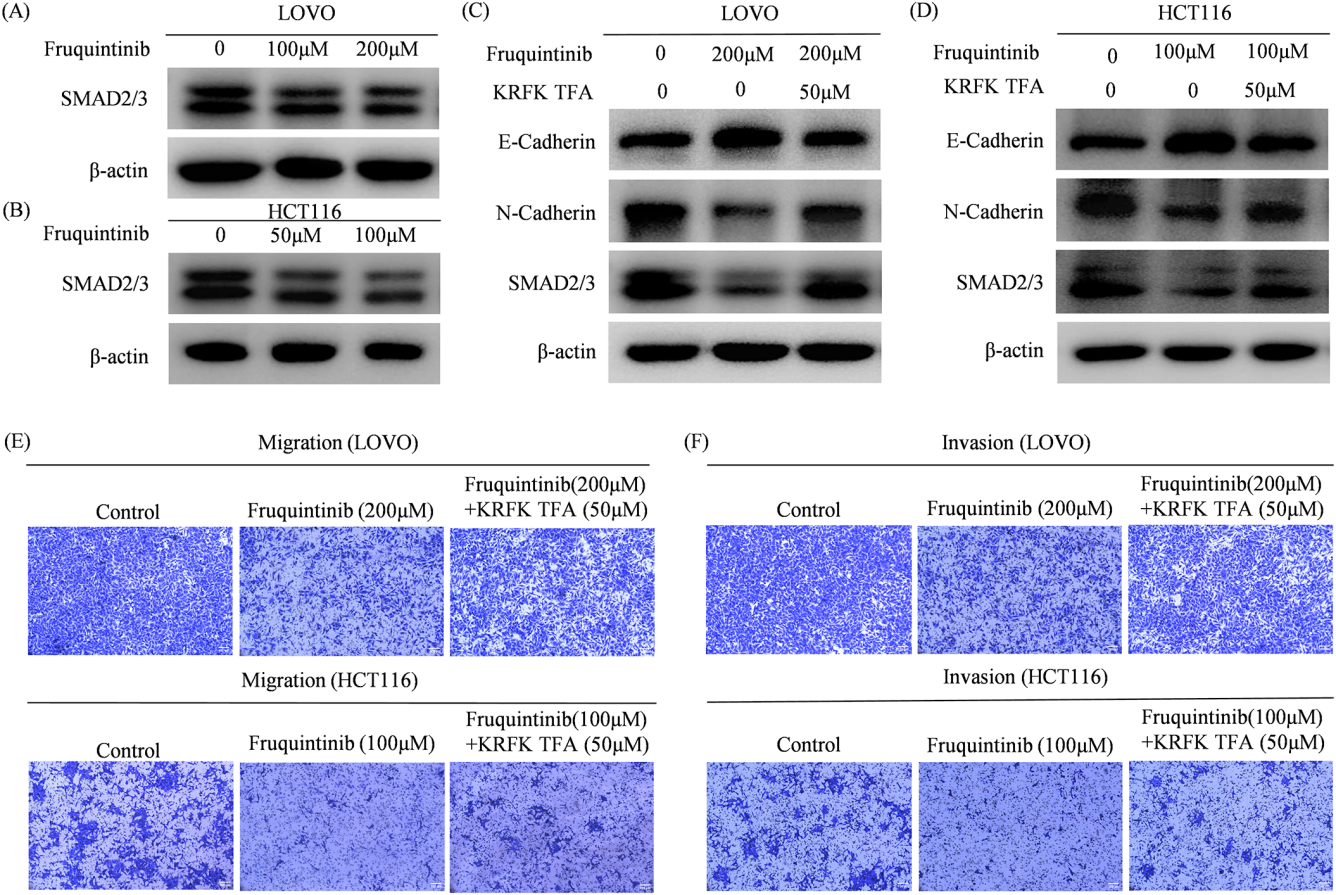
Fruquintinib inhibits the activation of EMT through the TGFβ-SMAD signal. The TGFβ receptor agonist KRFK TFA reverses the anti- migration and anti-invasion effect of fruquintinib. **(A, B)** SMAD2/3 protein expression in CRC cells after fruquintinib treatment. **(C, D)** SMAD2/3 protein expression treatment with fruquintinib or a combination of fruquintinib and KRFK TFA. **(E, F)** Cell migration and invasion determined by a Transwell assay in LOVO and HCT116 cells treated with fruquintinib or a combination of fruquintinib and KRFK TFA.

Our findings demonstrate that fruquintinib significantly curtails the migration and invasion of CRC cells, alongside a marked reduction in the expression of EMT-associated proteins. Given the critical role of the TGF-β/Smad pathway in regulating EMT (12), we employed western blotting to assess the expression of SMAD2/3 in CRC cells. The results revealed that fruquintinib markedly dampens SMAD2/3 expression in a dose-dependent manner (**Figures 7A, B**).

To confirm that fruquintinib exerts its inhibitory effects by modulating the TGFβ/SMAD2-3 signaling axis, we treated CRC cells with fruquintinib in conjunction with the TGF-β receptor agonist KRFK TFA. Western blotting analysis showed that KRFK TFA counteracted the fruquintinib-induced upregulation of E-cadherin and the downregulation of N-cadherin and SMAD2/3 (**Figures 7C, D**). Furthermore, transwell assays revealed that KRFK TFA mitigated fruquintinib’s suppressive effect on CRC cells (**Figures 7E, F**).

These findings suggest that KRFK TFA partially reverse inhibitory effect fruquintinib on CRC cell migration and invasion, highlighting the critical role of the TGF-β/Smad pathway in mediating fruquintinib’s mechanism of action.

### 3.6 The prediction of EMT-associated transcription factors

**Figure 8:**
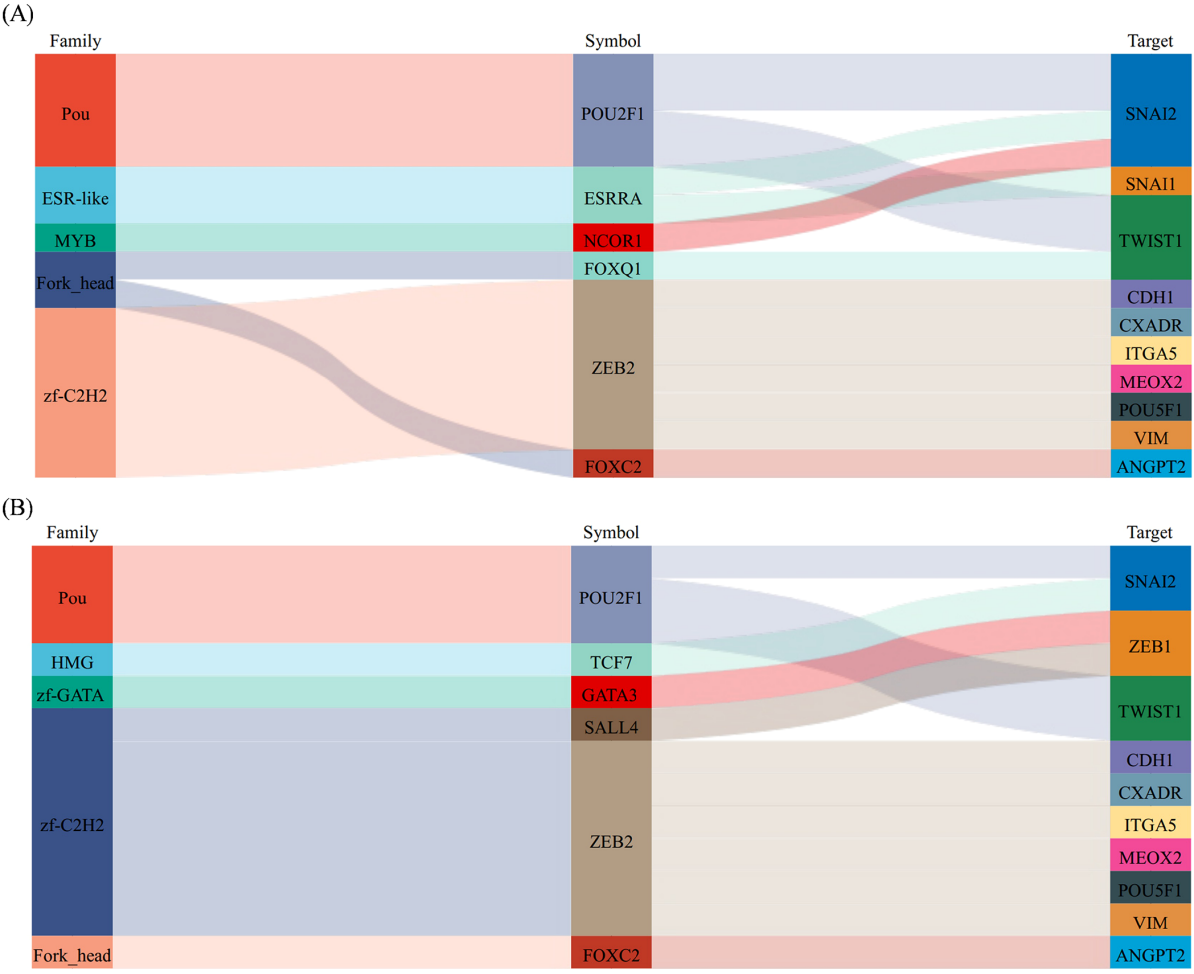
The prediction of EMT-inducing transcription factors (EMT-TFs). **(A, B)** Sankey diagram is used to show the correspondence between transcription factors and target factors. From left to right, the first column is the transcription factor family, the second column is transcription factor, and the third column is the target gene.

To further explore the changes in EMT, we predicted the differential expression of EMT-associated transcription factors (EMT-TFs) following fruquintinib treatment. Based on our differential expression analysis, we identified four key EMT-TFs: SNAI1, SNAI2, TWIST1, and ZEB2, which are well-known regulators of the EMT process (13). Among these transcription factors, ZEB2, SNAI1, and SNAI2 were found to target VIM (vimentin) and CDH1 (E-cadherin). Additionally, TWIST1 and ZEB1 were implicated in targeting other key EMT-associated genes. This conclusion was drawn based on bioinformatics analysis using the TRRUST2.0 database, with the regulatory relationships confirmed by Sangerbox3.0 software (**Figure 8**).

## 4 Discussion

Clinical studies have reported that 36%–81% of patients with metastatic CRC are diagnosed with multisite metastases (14), with 70-80% of these patients often classified as unresectable cancer, resulting in a survival rate of less than 5% (15). For CRC patients, conventional fluorouracil (FU)–based chemotherapy is the standard treatment with a median survival of 17-23 months (16). In recent years, the use anti-VEGF monoclonal antibodies has shown efficacy, extending median survival time to approximately 30 months in CRC patients; however, 5-year OS rates remain below 20% (3, 17). Tyrosine kinase inhibitors (TKIs), a new class of targeted antineoplastic agents, have dramatically improved patient survival and quality of life for various solid tumors (18). Fruquintinib, a tyrosine kinase inhibitor, has been reported to exert inhibitory effect on CRC, gastric cancer, lung cancer, advanced bone and soft tissue sarcoma cells (19–22). However, its precise mechanism has still not been fully elucidated.

To investigate the antitumor effect of fruquintinib in CRC, we conducted CCK-8 and colony formation assays, which demonstrated that this drug significantly inhibited the proliferation of CRC cells *in vitro* in a dose-dependent manner. A previous study has revealed that fruquintinib suppressed endothelial cell proliferation, inhibited tubule sprouting and prevented tumor angiogenesis (5). Additionally, fruquintinib has also been reported to inhibit proliferation and induce apoptosis in a mouse syngeneic model of CT26 cells (23). These findings are consistent with our *in vitro* results, suggesting that fruquintinib exerts its anti-tumor effects through multiple mechanisms, including inhibition of tumor cell proliferation and angiogenesis.

EMT plays a crucial role in colorectal cancer (CRC) progression, metastasis, and resistance to therapy. EMT is characterized by the loss of epithelial traits and the acquisition of mesenchymal properties, which enhances cancer cell migration and invasion (24, 25). Several key markers define E-cadherin is often downregulated in aggressive CRC, whereas mesenchymal markers such as N-cadherin, vimentin, and matrix metalloproteinases are upregulated, facilitating tumor invasion and metastasis (26). In CRC, the inverse relationship between the expression of E-cadherin and mesenchymal markers like N-cadherin and vimentin is associated with increased metastasis and poorer clinical prognosis (27–29). In our study, fruquintinib treatment led to the upregulation of E-cadherin and the downregulation of N-cadherin, vimentin, and MMP9, suggesting that it inhibits EMT and may reduce CRC cell motility and invasiveness. These results are consistent with findings for regorafenib, another TKI that inhibits EMT in cholangiocarcinoma and hepatocellular carcinoma (30, 31).

VEGF has been reported to play a crucial role in angiogenesis, vascular permeability and tumor cell invasion (32). Previous studies have shown that the EMT, characterized by the downregulation of E-cadherin, and the upregulation of vimentin and N-cadherin, promotes interstitial adhesion (33) and induces the expression of both VEGF and VEGFR-1 (34). Chen et al. found that VEGF regulated EMT markers (E-cadherin, N-cadherin and vimentin) (35). Fantozzi et al. found that the loss of E-cadherin in tumor cells correlated not only with increased tumor invasion and metastasis but also with increased VEGF-A-induced tumor angiogenesis (36). Moreover, the VEGF-A receptor neuropilin-1 has been shown to promote Snail nuclear localization, thereby enhancing EMT (37).

Fruquintinib is a potent VEGFR inhibitor, and emerging studies suggest that VEGF signaling interacts with the TGF-β pathway (38), which plays a pivotal role in epithelial-mesenchymal transition (EMT) and tumor progression. Our bioinformatics analysis also supported this hypothesis by identifying significant alterations in EMT-associated gene expression following fruquintinib treatment.

The TGF-β/Smad signaling pathway is a well-established regulator of EMT in CRC (39). SMAD2/3 phosphorylation is a key downstream event in TGF-β-induced EMT, leading to transcriptional changes that promote tumor progression (40). In fact, the overexpression of SMAD2 has been shown to negatively correlate with the expression of E-cadherin, contributing to poor prognosis in gastric cancer patients (41). Moreover, the suppression of E-cadherin can induce nuclear accumulation of SMAD2/3, increasing apoptosis (42). The SMAD3/4 complex, in association with SNAI1, represses E-cadherin expression and enhances N-cadherin expression, thereby promoting EMT in cancer cells (43, 44).

Our findings show that fruquintinib suppresses SMAD2/3 expression in a dose-dependent manner, indicating that it interferes with the TGF-β/Smad axis to prevent EMT activation. Moreover, the partial reversal of fruquintinib’s inhibitory effects on migration and invasion by KRFK TFA, a TGF-β receptor agonist, provided functional validation that the TGF-β/EMT axis is involved in fruquintinib’s mechanism of action. However, future research should further investigate this interplay in preclinical models to fully elucidate its therapeutic implications in colorectal cancer.

Given the central role of EMT markers and Smad signaling components in CRC, these molecules could potentially serve as predictive biomarkers for response to fruquintinib. Patients exhibiting high baseline expression of mesenchymal markers (e.g., N-cadherin, vimentin, MMP9) or activated Smad signaling may benefit from fruquintinib due to its EMT-suppressing effects. Conversely, monitoring changes in these markers post-treatment may help evaluate drug efficacy and guide personalized therapeutic strategies. However, further clinical studies are required to validate these biomarkers in patient cohorts and assess their predictive value in therapeutic outcomes.

In this study, we identified key EMT-associated transcription factors (EMT-TFs), including SNAI1, SNAI2, TWIST1, and ZEB2, based on bioinformatic predictions derived from differential gene expression analysis. These predictions were supported by established transcription factor databases (such as AnimalTFDB, Pfam, and DBD) and further analyzed using the TRRUST2.0 database to infer potential regulatory relationships between these transcription factors and their target genes, such as VIM (vimentin) and CDH1 (E-cadherin). The key members of EMT-TFs, including ZEB, SNAIL and TWIST, play critical roles in orchestrating the extensive transcriptional changes associated with EMT (45–47). Thiery et al. reported that SNAIL and ZEB directly repressed E-cadherin. While Twist acted as an indirect E-cadherin repressor. Notably, Snai1 upregulates the expression of Zeb family proteins in carcinoma cells by inducing the expression of a natural antisense transcript for ZEB2 (NAT) (48). Zheng et al. reported that ZEB2 and TWIST1 expression levels are elevated in CRC tissues and positively correlated with CRC stages. Moreover, ZEB2 can recruit TWIST1, PRMT5, and NuRD complex to form a repressive multicomplex that epigenetically silences CDH1, thereby driving EMT process and promoting metastasis in CRC (49). These findings further underscore the potential of fruquintinib to modulate EMT-TFs and suppress EMT-associated metastasis in CRC.

Although bioinformatics approaches provide valuable insights and are widely used in the field, we acknowledge that these findings are based on computational predictions rather than experimental validation. Our study has limitations in confirming the direct interactions between the identified EMT-TFs and their target genes. Further experimental validation, such as qRT-PCR and ChIP assays, would be necessary to definitively confirm these predicted regulatory relationships. This represents an important avenue for future research to strengthen the mechanistic understanding of fruquintinib’s effects on EMT in CRC.

While our study provides significant insights into the molecular mechanisms of fruquintinib in CRC, it is essential to consider how these *in vitro* findings translate into *in vivo* models and clinical outcomes. Our results demonstrate that fruquintini effectively inhibits CRC cell migration and invasion by suppressing the epithelial-mesenchymal transition (EMT) via the TGF-β/Smad pathway. Given that EMT is a key driver of CRC metastasis (50), these findings suggest that fruquintinib may exhibit anti-metastatic properties *in vivo* by maintaining epithelial characteristics and preventing tumor cell dissemination. However, we acknowledge that our investigation was conducted solely *in vitro*. As fruquintinib is already FDA-approved for metastatic CRC (51), further studies utilizing *in vivo* metastatic models are necessary to comprehensively evaluate its mechanistic effects within a physiological tumor microenvironment. Future research should focus on validating our findings in animal models to explore the systemic impact of fruquintinib on EMT regulation and metastatic progression. These studies will be crucial in bridging the gap between our *in vitro* results and clinical applicability.

Clinically, fruquintinib has demonstrated efficacy as a third-line treatment for metastatic CRC (52). Our findings suggest that tumors with high EMT activity may be more responsive to fruquintinib due to its ability to reverse mesenchymal traits and suppress invasion. Therefore, assessing EMT and Smad signaling markers in patient-derived tumor samples may help guide personalized treatment strategies and improve therapeutic outcomes. However, further clinical validation is required to establish the predictive value of these biomarkers in CRC patients receiving fruquintinib treatment.

Despite the need for additional *in vivo* and clinical studies, our findings provide a foundation for further mechanistic research and highlight the potential of fruquintinib as a targeted therapy in CRC, particularly in cases with active EMT-driven progression.

## 5 Conclusion

Fruquintinib effectively inhibits the migration and invasion of CRC cells by disrupting the EMT process through the TGF-β-Smad signaling pathway. These findings highlight the potential of fruquintinib as a therapeutic agent in CRC treatment, highlighting the need for further investigation into its clinical applicability.

## Data Availability

The datasets presented in this study can be found in online repositories. The names of the repository/repositories and accession number(s) can be found below: https://ngdc.cncb.ac.cn/gsa-human, HRA005402.
