# Supplementary figures and images for: Fruquintinib inhibits the migration and invasion of colorectal cancer cells by modulating epithelial-mesenchymal transition via TGF-β/Smad signaling pathway
Source: Front Oncol. 2025 Mar 11;15:1503133. doi: 10.3389/fonc.2025.1503133 (PMC11932892; doi:10.3389/fonc.2025.1503133)

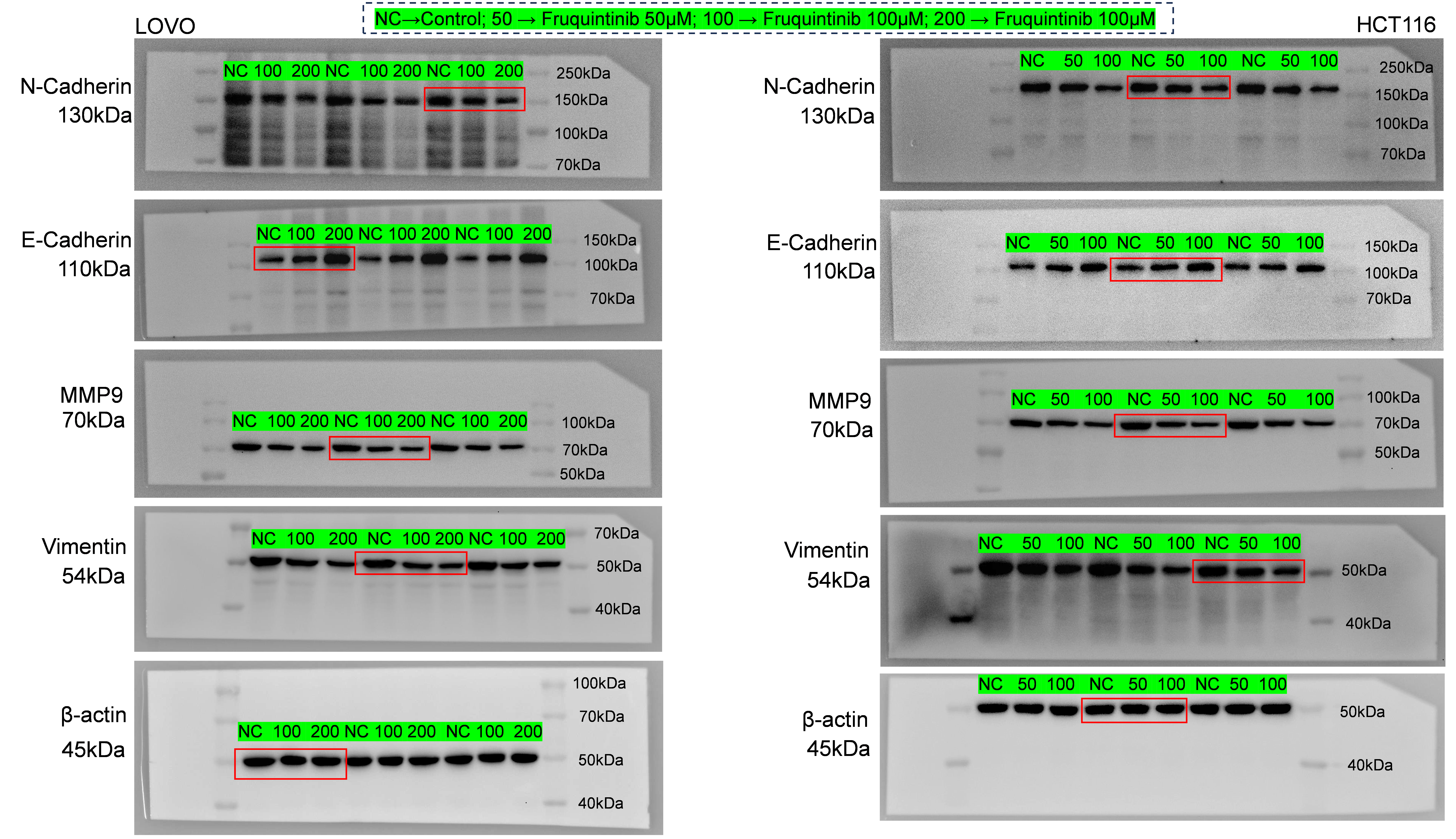

Supplement: Supplementary file 2 [file Image1.tif]

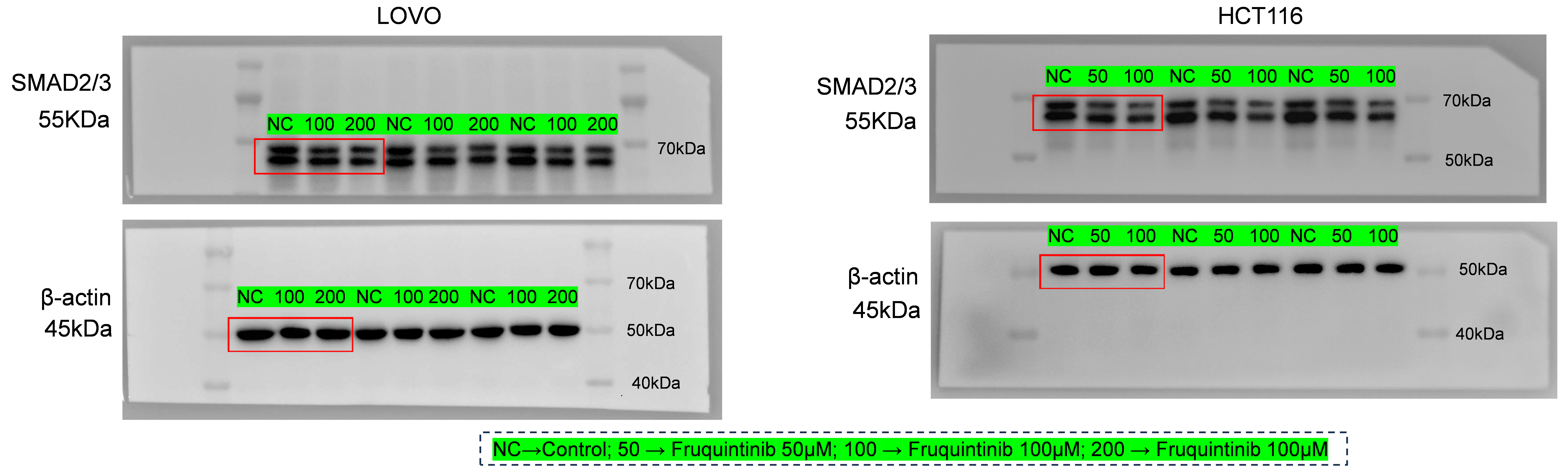

Supplement: Supplementary file 3 [file Image2.tif]

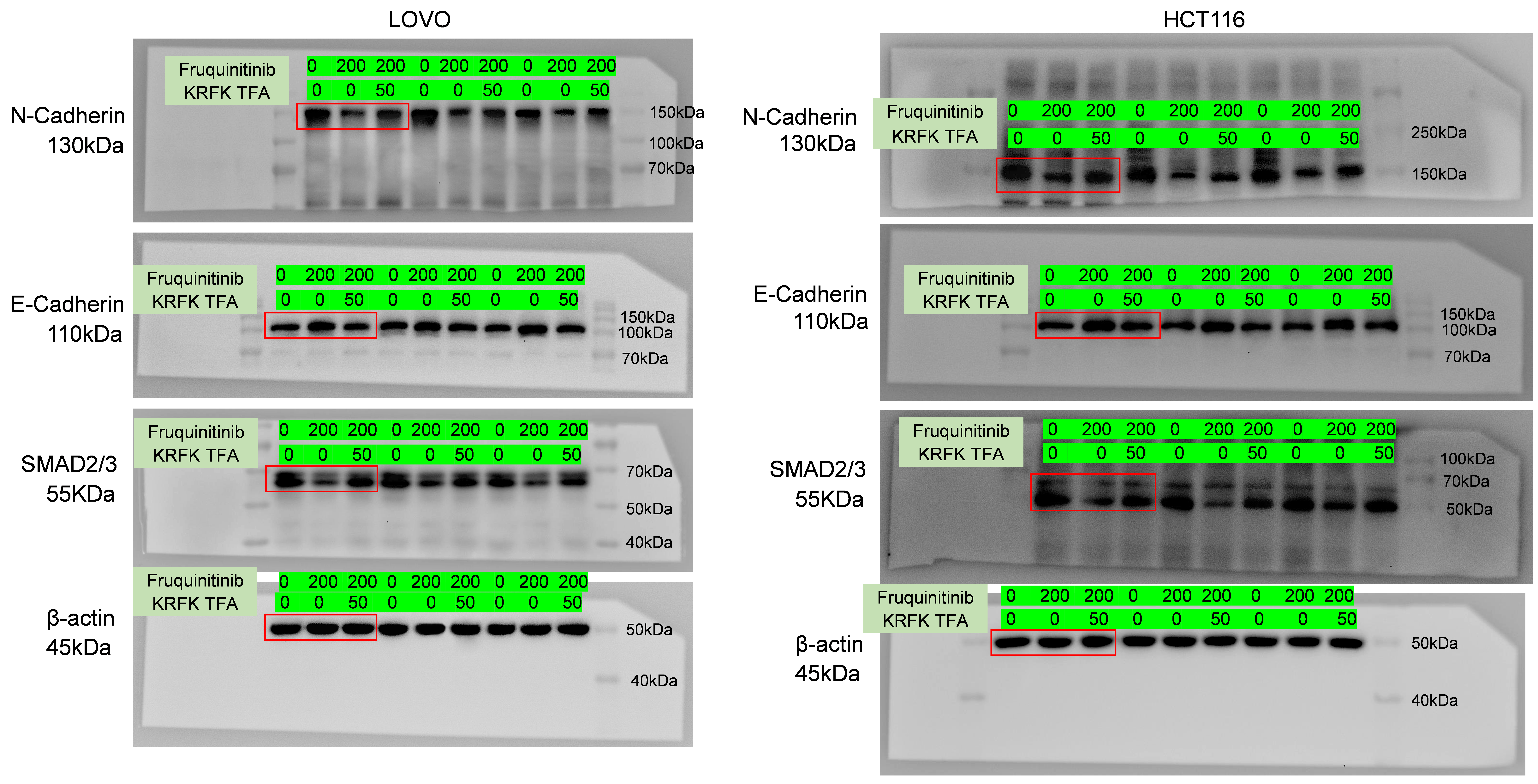

Supplement: Supplementary file 4 [file Image3.tif]
